# MiR-324-5p assists ultrasonography in predicting lymph node metastasis of unifocal papillary thyroid microcarcinoma without extracapsular spread

**DOI:** 10.18632/oncotarget.19717

**Published:** 2017-07-31

**Authors:** Yanhua Yang, Shujun Xia, Xiaofeng Ni, Zhongxin Ni, Lu Zhang, Wenhan Wang, Yanjun Kong, Yan Wang, Lei Ye, Weiwei Zhan

**Affiliations:** ^1^ Department of Obstetrics and Gynecology, Ruijin Hospital, Shanghai Jiao Tong University School of Medicine, Shanghai, China; ^2^ Department of Ultrasonography, Ruijin Hospital, Shanghai Jiao Tong University School of Medicine, Shanghai, China; ^3^ Department of Endocrinology and Metabolism, Ruijin Hospital, Shanghai Jiao Tong University School of Medicine, Shanghai, China

**Keywords:** papillary thyroid carcinoma, lymph node metastasis, miR-324-5p, ultrasonography, fine needle aspiration

## Abstract

Ultrasonography is the first choice of lymph node metastasis (LNM) detection which is crucial for therapeutic options of papillary thyroid cancer (PTC). However, the sensitivity of ultrasonography in detecting LNM of PTC is relatively low; especially in central LNM. MiR-324-5p has been reported to play important roles in the metastasis of various cancers. To explore the relationship between miR-324-5p and LNM in PTC, quantitative real-time polymerase chain reaction was performed in PTC tissue and fine needle aspiration (FNA) washout successively. Its correlation with LNM of PTC was analyzed. The clinicopathological and sonographic factors relating to LNM were also studied. Additionally, the function assay of miR-324-5p in PTC cells was conducted. Current study demonstrated that age was an independent protective factor and multifocality, advanced TNM stage, increased transverse diameter of thyroid nodule, ultrasound suspected LNM were independent risk factors of LNM. MiR-324-5p promoted proliferation, migration and invasion of PTC cell line. MiR-324-5p could serve as a candidate predictor along with ultrasonography in predicting LNM, especially central LNM of unifocal papillary thyroid microcarcinoma without extracapsular spread.

## INTRODUCTION

Papillary thyroid cancer (PTC) is the most common type which accounts for 85% to 90% of all thyroid cancers [1–5]. In general, PTC is an indolent cancer with a high curability, but a subgroup of PTCs maintain aggressive feature with a poor prognosis [6, 7]. Risk stratifications would be of great value to improve the management of PTC.

Lymph node metastasis (LNM) is an indicator of PTC recurrence and is directly related to the extent of thyroid cancer surgery [8–11]. Reoperation will bring economic burden and high incidence of complication to patients. Radioiodine (RAI) therapy may be not effective in cases that do not have adequate radioiodine uptake (131I-refractory) or have 18-fluorodeoxyglucose positron emission tomography (18FDG-PET) positive metastases [11]. Preoperative 18FDG-PET scanning is not routinely recommended [12]. Ultrasound (US) is a valuable tool for thyroid cancer screening as well as LNM detection [12–16]. However, due to its low sensitivity in the central neck, the role of US in surgical planning for central compartment neck dissection in PTC is constrained [15, 17, 18]. So is computed tomography (CT) [17]. Therefore, it is pivotal to find out an effective molecular marker to assist in LNM prediction, especially in cN0 patients if such data would be expected to alter initial surgical decision making and achieve personalized treatment..

In recent years, studies of microRNAs (miRNAs) as potential biomarkers for diagnosis, prognosis, or therapeutic targets of cancer have drawn great attention [19, 20]. As one of the gene expression regulators, miRNAs are found to negatively modulate gene expression by targeting the 3’ untranslated region of mRNAs in a sequence-specific manner, and are involved in diverse biological processes [21–23]. Among them, miR-324-5p has been proven to be involved in the growth, invasion, and migration of different kinds of cancer [24–28]. However, its role in LNM of PTC remains unknown. Fine needle aspiration (FNA) is a high cost-effective method in PTC assessment [29–31]. It has been reported that detection of miRNA or BRAF mutation in FNA washout is helpful in estimating the character or invasiveness of thyroid nodules [32, 33]. Hence, the study aimed to investigate the role of miR-324-5p in PTC and the value of FNA-miR-324-5p together with ultrasonography in predicting LNM of PTC.

## RESULTS

### Clinicopathological characteristics of PTC subjects

The clinicopathological characteristics of 41 PTC tissues and 143 FNA samples are summarized in Tables 1 and 2. All the subjects were divided into two groups according to the pathologic findings: LNM (-) and LNM (+). None of the cases presented with distant metastasis. As shown in Table 2, in LNM (+) group, there were more patients with young age, multi-foci, extracapsular spread (ECS) and advanced TNM stage (III/IV) compared to LNM (-) group.

**Table 1:** Clinicopathological characteristics of PTC tissues

	Total	LNM (-)	LNM (+)	P value
Sex (n,%)				
Male	8 (19.5%)	3	5	0.992
Female	33 (80.5%)	15	18	
Age (y)	46.27±10.86	47.00±9.17	45.70±12.19	0.708
<45 y (n,%)	18 (43.9%)	9	9	0.486
≥45 y (n,%)	23 (56.1%)	9	14	
Size (mm) ^*^	12.0 (7.5-15.0)	9.5 (6.8-15.8)	12.0 (8.0-15.0)	0.342
Folcality (n,%)				
Unifocal	25 (61.0%)	13	12	0.192
Multifocal	16 (39.0%)	5	11	
ECS (n,%)				
No	29 (70.7%)	14	15	0.380
Yes	12 (29.3%)	4	8	
TNM stage (n,%)				
I-II stage	25 (61.0%)	15	10	0.009
III-IV stage	16 (39.0%)	3	13	

^*^ Quartile for non-normal distribution data. ECS: extracapsular spread.

**Table 2:** Clinicopathological characteristics of FNA samples

	Total	LNM (-)	LNM (+)	P value
Sex (n,%)				
Male	33 (23.1%)	17 (20.2%)	16 (27.1%)	0.336
Female	110 (76.9%)	67 (79.8%)	43 (72.9%)	
Age (y) ^*^	40 (34-51)	43 (35-54)	37 (30-47)	**0.003**
<45 y (n,%)	82 (57.3%)	45 (53.6%)	37 (62.7%)	0.277
≥45 y (n,%)	61 (42.7%)	39 (46.4%)	22 (37.3%)	
Folcality (n,%)				**0.010**
Unifocal	108 (75.5%)	70 (83.3%)	38 (64.4%)	
Multifocal	35 (24.5%)	14 (16.7%)	21 (35.6%)	
ECS (n,%) ^a^				**0.030**
No	120 (85.1%)	76 (90.5%)	44 (77.2%)	
Yes	21 (14.9%)	8 (9.5%)	13 (22.8%)	
TNM stage (n,%)				**<0.001**
I-II stage	118 (82.5%)	81 (96.4%)	37 (62.7%)	
III-IV stage	25 (17.5%)	3 (3.6%)	22 (37.3%)	

^*^ Quartile for non-normal distribution data. ^a^ Two cases of Tx. FNA: fine needle aspiration; ECS: extracapsular spread.

### Sonographic characteristics of FNA samples

All the FNA nodules were markedly hypoechoic or hypoechoic with solid or mostly solid architecture. Nodules with LNM were more likely to be located throughout the internal and external of thyroid lobe than those without LNM (16.1% vs.3.6%, P=0.023). The median of greatest dimension was 7.5mm (range 2.4-44mm) and PTMC made up for 65.7%. As summaried in Table 3, nodule size in LNM (-) group was smaller compared to LNM (+) group and LNM (-) group contained more PTMC (P<0.001). A/T≥1, microcalcification, ultrasound suspected LNM (US-LNM) was significantly correlated with LNM. However, no significant associations were observed between A/L≥1, border, amount of color Doppler signals and LNM.

**Table 3:** Sonographic characteristics of FNA samples

	Total	LNM (-)	LNM (+)	P value
Greatest dimension (mm) ^*^	7.5 (5.2-12.4)	6.5 (4.7-8.3)	12.1 (6.6-17.0)	**<0.001**
L (mm) ^*a^	7.0 (5.0-11.8)	6.0 (4.4-8.3)	11.3 (6.2-17.0)	**<0.001**
A (mm) ^*^	6.3 (4.6-9.4)	5.4 (4.4-7.0)	8.3 (5.2-12.0)	**<0.001**
T (mm) ^*b^	6.4 (4.6-10.3)	5.4 (4.1-7.4)	9.5 (5.9-14.2)	**<0.001**
A/L≥1 (n,%) ^a^				0.167
Yes	46 (32.4%)	31 (36.9%)	15 (25.9%)	
No	96 (67.6%)	53 (63.1%)	43 (74.1%)	
A/T≥1 (n,%) ^b^				**0.010**
Yes	70 (49.3%)	49 (58.3%)	21 (36.2%)	
No	72 (50.7%)	35 (41.7%)	37 (63.8%)	
PTMC (n,%)				**<0.001**
Yes	94 (65.7%)	70 (83.3%)	24 (40.7%)	
No	49 (34.3%)	14 (16.7%)	35 (59.3%)	
Microcalcification (n,%)				**0.040**
Yes	80 (55.9%)	41 (48.8%)	39 (66.1%)	
No	63 (44.1%)	43 (51.2%)	20 (33.9%)	
Margin (n,%)				0.630
Regular	7 (4.9%)	3 (3.6%)	4 (6.8%)	
Irregular	136 (95.1%)	81 (96.4%)	55 (93.2%)	
Color Doppler signal (n,%)				0.209
None/low	98 (68.5%)	61 (72.6%)	37 (62.7%)	
Medium/high	45 (31.5%)	23 (27.4%)	22 (37.3%)	
US-LNM (n,%)				**<0.001**
Yes	24 (16.8%)	6 (7.1%)	18 (30.5%)	
No	119 (83.2%)	78 (92.9%)	41 (69.5%)	

^*^ Quartile for non-normal distribution data. ^a^ One nodule adjacent to the isthmus was not quoted. ^b^ One nodule in the isthmus was not quoted. A: anteroposterior diameter; L: longitudinal diameter; T: transverse diameter; US-LNM: ultrasound suspected LNM.

### Clinicopathological and sonographic factors associated with LNM

Univariate analysis illuminated that increased age, A/T≥1, PTMC were protective factors of LNM. Multi-foci, ECS, advanced stage, location throughout the internal and external of thyroid lobe, increased dimension, microcalcification, US-LNM were risk factors of LNM. Multivariate logistic regression demonstrated that increased age was an independent protective factor of LNM. Multi-foci, increased transverse diameter, advanced stage and US-LNM were independent risk factors of LNM (Table 4).

**Table 4:** Uni- and multi-variate regression analysis of factors associated with LNM

Variate	Univariate analysis		Multivariate analysis	
	**OR (95% CI)**	**P value**	**OR (95% CI)**	**P value**
Age (y)	0.952 (0.921-0.984)	0.003	0.850 (0.780-0.926)	<0.001
Multifocality	2.763 (1.263-6.047)	0.011	6.394 (1.778-22.993)	0.004
ECS	2.807 (1.079-7.300)	0.034		
III-IV stage	16.054 (4.520-57.019)	<0.001	186.914 (23.125-1510.767)	<0.001
Location^*^	5.170 (1.333-20.047)	0.017		
Greatest dimension (mm)	1.218 (1.117-1.327)	<0.001		
L (mm)	1.213 (1.113-1.321)	<0.001		
A (mm)	1.296 (1.154-1.457)	<0.001		
T (mm)	1.274 (1.148-1.413)	<0.001	1.245 (1.090-1.423)	0.001
A/T≥1	0.405 (0.204-0.808)	0.010		
PTMC	0.137 (0.063-0.297)	<0.001		
microcalcification	2.045 (1.028-4.070)	0.042		
US-LNM	5.707 (2.103-15.488)	0.001	7.142 (1.583-32.219)	0.011

^*^ Located throughout the internal and external of thyroid lobe. ECS: extracapsular spread ; A: anteroposterior diameter; L: longitudinal diameter; T: transverse diameter; US-LNM: ultrasound suspected LNM; OR : odds ratio; CI : confidence interval.

### The expression of miR-324-5p in PTC subjects and cell line

**Figure 1:**
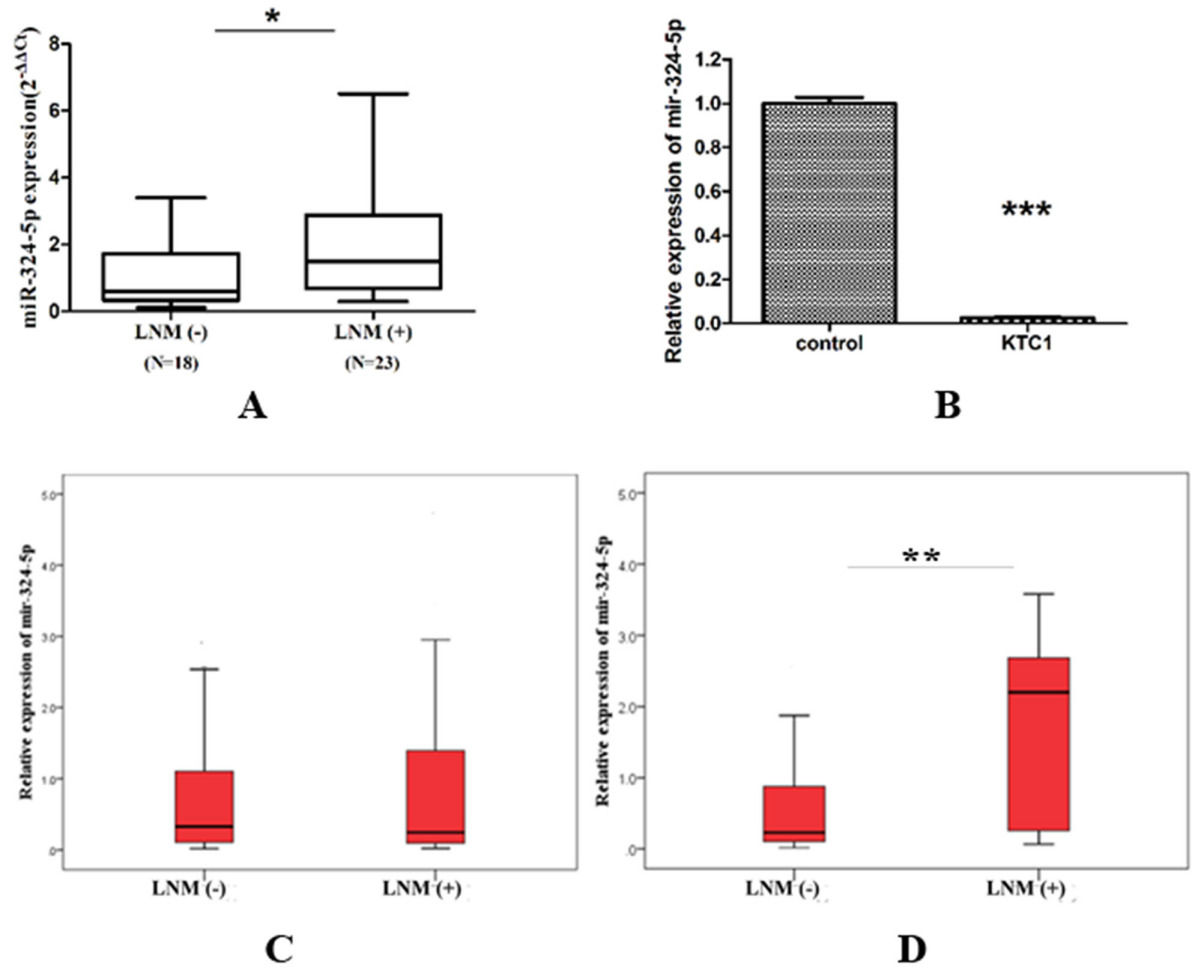
Relative expression of miR-324-5p in PTC subjects and cell line **(A)** PTC tissues (41 cases). Data are represented as quartile. ^*^p<0.05. **(B)** KTC1 cell line. Data are expressed as means ± SD. ^***^P<0.001. **(C)** Total FNA washout specimens (143 cases). Data are represented as quartile. **(D)** FNA washout of unifocal PTMC without ECS (66 cases). Data are represented as quartile. ^**^p<0.01.

As depicted in Figure 1A, miR-324-5p was significantly overexpressed in group LNM (+) compared with group LNM (-) [1.50 (0.69-2.87) vs. 0.61 (0.34-1.72), p=0.026] in 41 PTC tissues. The expression of miR-324-5p was significantly lower in KTC1 cell line than in control (PTC without LNM) (1.000±0.027 vs. 0.027±0.001, P<0.001) (Figure 1B).

As denoted in Figure 1C, the expression of miR-324-5p was not significantly different between LNM (-) group and LNM (+) group in 143 FNA samples. However, among 66 unifocal PTMC without ECS, miR-324-5p was overexpressed in LNM (+) group compared with LNM (-) group [2.20 (0.23-2.81) vs. 0.23 (0.10-0.90) P=0.009] (Figure 1D).

### Characteristics and LNM prediction of unifocal PTMC without ECS

In the light of the pathologic finding, unifocal PTMC without ECS were divided into LNM (-) group (n=54) and LNM (+) group (n=12) (Table 5). Advanced TNM stage and higher miR-324-5p level were correlated with LNM.

**Table 5:** Characteristics of unifocal PTMC without ECS

	LNM (-)	LNM (+)	P value
Gender (n,%)			0.304
Male	12 (22.2%)	5 (41.7%)	
Female	42 (77.8%)	7 (58.3%)	
Age (y)^*^	42 (35-54)	36 (30-46)	0.074
TNM stage (n,%)			**0.001^a^**
I-II stage	54 (100.0%)	8 (66.7%)	
III-IV stage	0 (0.0%)	4 (33.3%)	
Greatest dimension (mm)	6.0±1.9	6.7±1.8	0.236
L (mm)	5.6±1.9	6.6±1.8	0.093
A (mm)	5.1±1.4	5.5±1.3	0.328
T (mm)	5.0±1.6	5.7±1.4	0.163
A/L≥1 (n,%)			0.492
Yes	22 (40.7%)	3 (25.0%)	
No	32 (59.3%)	9 (75.0%)	
A/T≥1 (n,%)			0.701
Yes	33 (61.1%)	6 (50.0%)	
No	21 (38.9%)	6 (50.0%)	
Microcalcification (n,%)			0.322
Yes	23 (42.6%)	7 (58.3%)	
No	31 (57.4%)	5 (41.7%)	
US-LNM (n,%)			0.203
Yes	4 (7.4%)	3 (25.0%)	
No	50 (92.6%)	9 (75.0%)	
MiR324-5p expression			**<0.001**
<2.01	51 (94.4%)	5 (41.7%)	
>2.01	3 (5.6%)	7 (58.3%)	

^*^ Quartile for non-normal distribution data. ^a^ Fisher's exact test. ECS: extracapsular spread; A: anteroposterior diameter; L: longitudinal diameter; T: transverse diameter; US-LNM: ultrasound suspected LNM.

**Figure 2:**
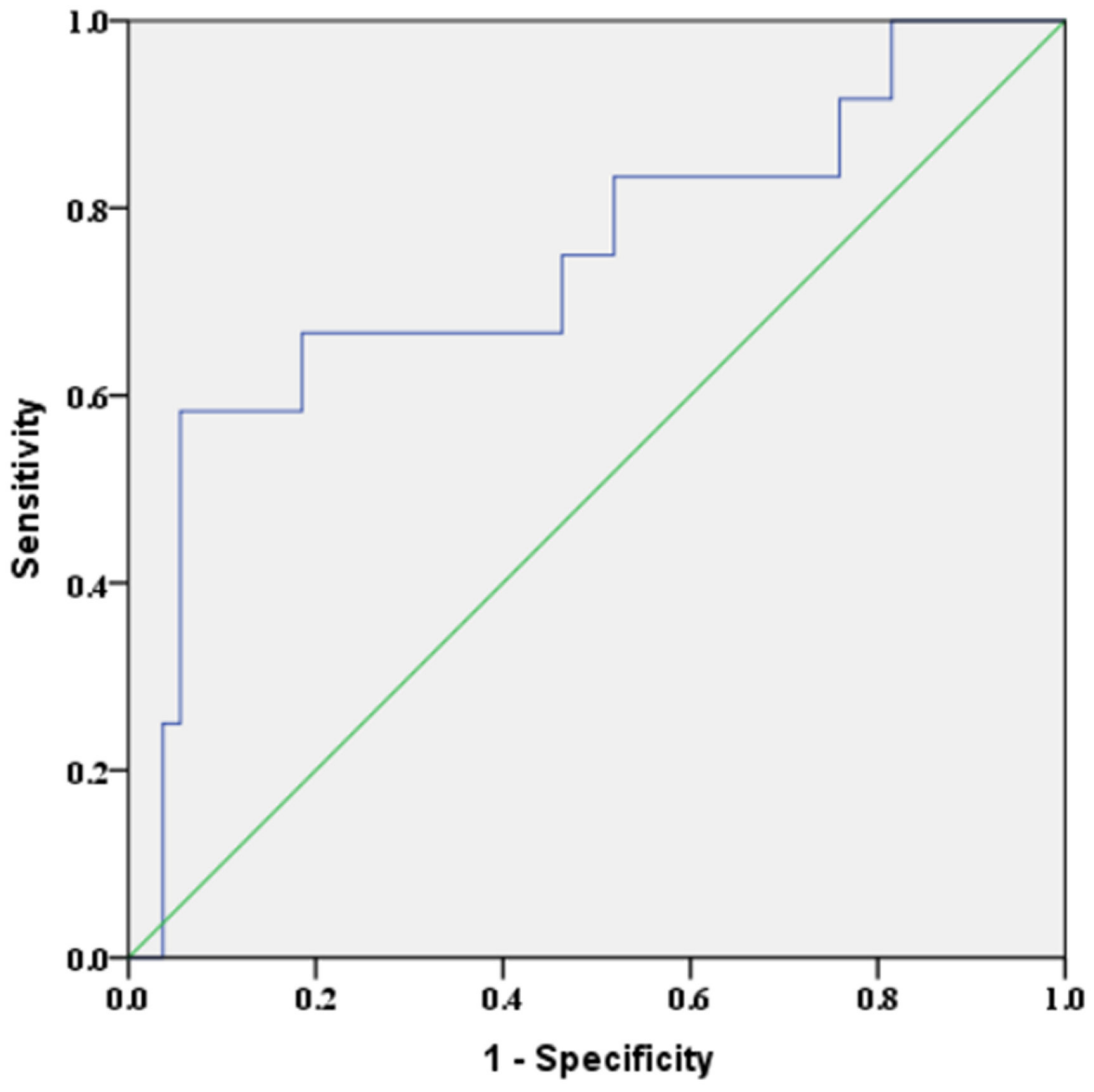
ROC curve of miR-324-5p for distinguishing LNM in unifocal PTMC without ECS AUC=0.744 (95%CI: 0.571-0.917), p=0.009.

Receiver operator characteristic (ROC) curve analysis was performed in these particular subjects, area under the curve (AUC) of miR-324-5p to distinguish LNM was 0.744 (95%CI: 0.571-0.917) (Figure 2). The optimal cut-off value was 2.01. The corresponding sensitivity, specificity, PPV and NPV was 58.33%, 94.44%, 70.00% and 91.07%, which was all higher than that of US-LNM (Table 6). Nine LNM cases missed diagnosed by ultrasound were all central LNM. Among six of them, miR-324-5p relative expression was >2.01. Subgroup ‘US-LNM or miR-324-5p>2.01’ harboured the highest sensitivity of 75.00% in predicting LNM of PTC.

**Table 6:** Diagnostic value of miR-324-5p and US-LNM in LNM prediction of unifocal PTMC without ECS

	Sensitivity	Specificity	PPV	NPV	Accuracy
MiR-324-5p>2.01	58.33%	94.44%	70.00%	91.07%	87.88%
US-LNM	25.00%	92.59%	42.86%	84.75%	80.30%
US-LNM or miR-324-5p>2.01	75.00%	87.04%	56.25%	94.00%	84.85%

US-LNM: ultrasound suspected LNM; ECS: extracapsular spread; NPV: negative predictive value; PPV : positive predictive value.

### MiR-324-5p promotes the proliferation and inhibits the apoptosis of KTC1 cells

**Figure 3:**
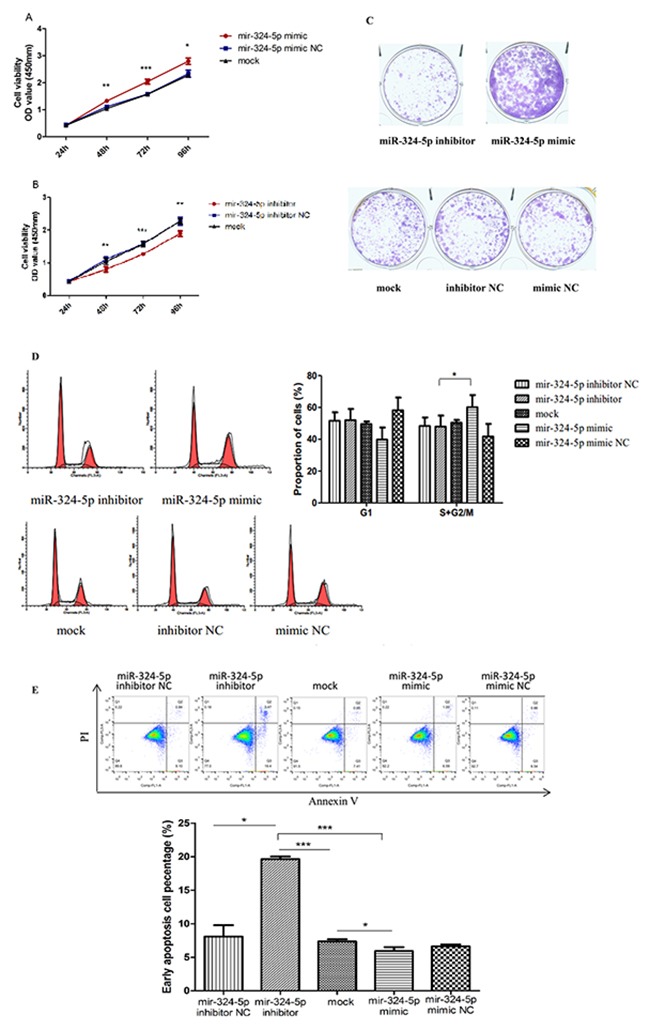
MiR-324-5p promoted proliferation and inhibited apoptosis of KTC1 cells **(A-B)** Cell proliferation assay in KTC1 transfected with miR-324-5p mimic (A) and inhibitor (B). **(C)** Colony formation assay of KTC1 transfected with miR-324-5p inhibitor, mimic, mock, inhibitor NC and mimic NC(×100). **(D)** Cell cycle of KTC1 transfected with miR-324-5p inhibitor, mimic, mock, inhibitor NC and mimic NC. **(E)** Apoptosis assay of KTC1 transfected with miR-324-5p inhibitor, mimic, mock, inhibitor NC and mimic NC. Data are expressed as means ± SD of three independent experiments. ^*^p<0.05, ^**^p<0.01, ^***^P<0.001.

KTC1 was transfected with miR-324-5p mimic, miR-324-5p mimic scramble (mimic NC), miR-324-5p inhibitor, miR-324-5p inhibitor scramble (inhibitor NC) and mock-vehicle. Then we utilized CCK8 analysis and colony formation assay to evaluate the effect of miR-324-5p on cell proliferation. As shown in Figure 3A, 3B, miR-324-5p upregulation significantly induced cell viability at 48h, 72h, and 96h, while miR-324-5p downregulation significantly inhibited cell viability at 48h, 72h, and 96h. Corresponding to that, colony formation assay indicated that there were more colonies in miR-324-5p mimic group compared with inhibitor group and control group (Figure 3C).

Cell cycle analysis revealed that the proliferation index (i.e.: the proportion of cells in the S+G2/M phase) was significantly higher in mimic group than in inhibitor group, while the proportion of cells in the G1 phase had no statistical difference among five groups (Figure 3D).

Apoptosis assay indicated that miR-324-5p upregulation inhibited apoptosis, while miR-324-5p downregulation enhanced apoptosis in KTC1. (Figure 3E).

Taken these results together, miR-324-5p was capable of promoting KTC1 cell proliferation by increasing proliferation index and suppressing apoptosis.

### MiR-324-5p promotes the invasiveness and migration of KTC1 cells

**Figure 4:**
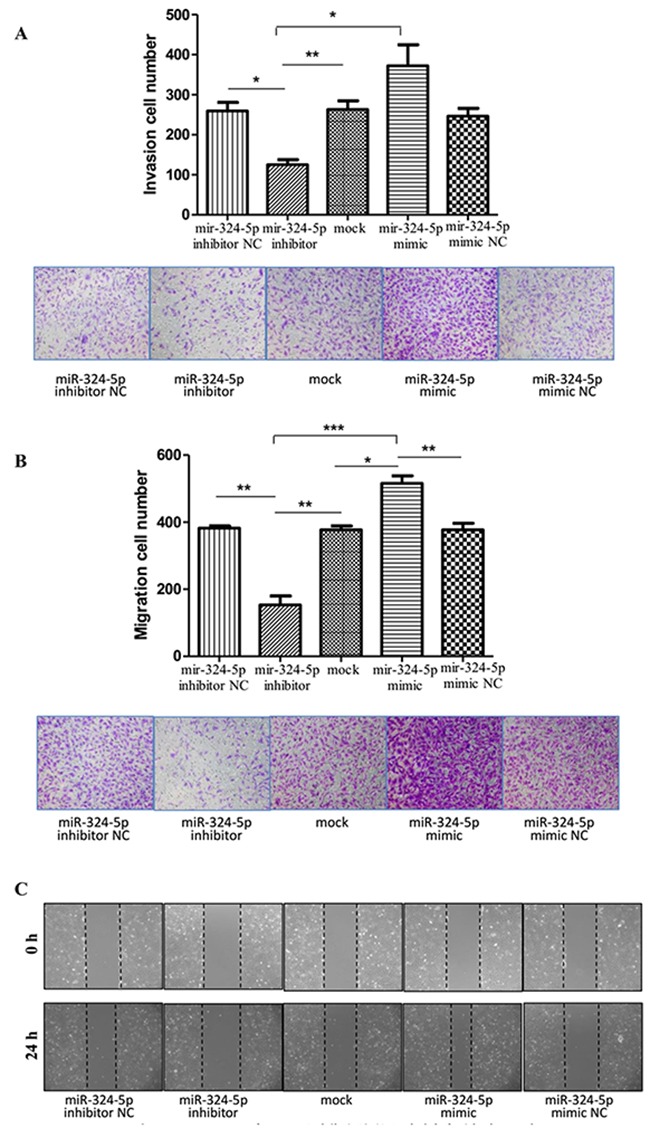
MiR-324-5p promoted invasiveness and migration of KTC1 cells **(A)** Transwell invasion assay of KTC1 transfected with miR-324-5p inhibitor, mimic, mock, inhibitor NC and mimic NC (×100). **(B)** Transwell migration assay of KTC1 transfected with miR-324-5p inhibitor, mimic, mock, inhibitor NC and mimic NC (×100). **(C)** Wound healing assay of KTC1 transfected with miR-324-5p inhibitor, mimic, mock, inhibitor NC and mimic NC (×100). Data are expressed as means ± SD of three independent experiments. ^*^p<0.05, ^**^p<0.01, ^***^P<0.001.

Transwell invasion assay showed that the invading cells were significantly fewer in miR-324-5p inhibitor group (125.33 ± 12.42) than in miR-324-5p mimic group (372.33 ± 52.27) as well as in miR-324-5p inhibitor NC and mock-vehicle group (Figure 4A).

According to the transwell migration assay, the number of cells that migrated through the chamber was significantly higher in miR-324-5p mimic group (515.67 ± 22.81) than in miR-324-5p inhibitor group (153.33 ± 26.31) as well as in miR-324-5p mimic NC and mock-vehicle group (Figure 4B). The similar trend was observed in the scratch assay (Figure 4C).

Data above indicated that miR-324-5p promoted invasiveness and migration of KTC1 cells.

## DISCUSSION

US-LNM was an independent risk factor of LNM. However, the sensitivity of US detected LNM in unifocal PTMC without ECS was only 25.00%. The results emphasize the necessity of a specific marker complementary to US. Among these particular subjects, miR-324-5p was overexpressed in LNM (+) group and the diagnostic value of miR-324-5p>2.01 was all higher than that of US-LNM. The sensitivity reached the highest of 75.00% when miR-324-5p>2.01 and ultrasonography were combined for LNM prediction. Moreover, LNM cases missed diagnosed by US were all in central compartment. MiR-324-5p levels in two thirds of those cases were higher than the critical value 2.01. Furthermore, factors which can be obtained preoperatively were not correlated with LNM of unifocal PTMC without ECS, except for miR-324-5p. *In vitro* functional assay also elucidates that miR-324-5p promotes proliferation, migration and invasiveness of PTC cells and inhibits apoptosis as well. Thus, we regard miR-324-5p as a potential biomarker that assists ultrasonography in LNM, especially central LNM prediction in unifocal PTMC without ECS.

Features such as age, sex multifocality, size and location have been reported to be highly associated with LNM in cN0 patients [34]. In our study, these clinical features were significantly associated with LNM by univariate analysis except for sex. Age was an independent protective factor of LNM, indicating that younger patients are prone to LNM, which is in line with the other studies [18, 35–39]. Increased transverse diameter was an independent risk factor of LNM. The proportion of nodules of A/T<1 was significantly higher in LNM (+) group. So was the proportion of nodules located throughout the internal and external of thyroid lobe. These results hint that LNM is more common in nodules with lateral growth, which is consistent with Xu's results [35, 40]. Current study didn't show any correlation between amount of color Doppler signals and LNM. This might be because it is hard to detect color Doppler signals in PTMC, which accounted for 65.7% of the total PTC in this study.

**Figure 5:**
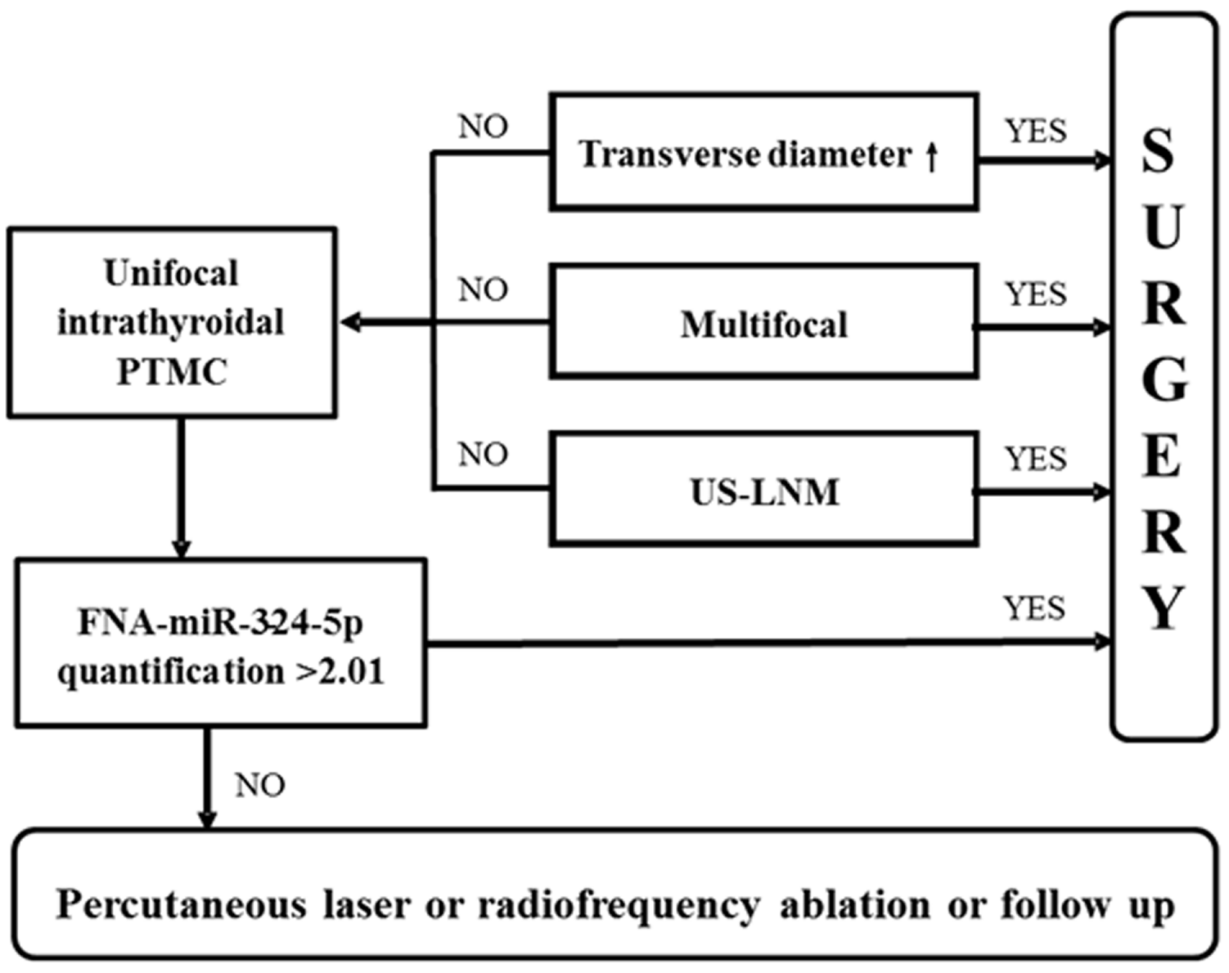
LNM assessment flow chart in PTC

ECS was a risk factor of LNM. Multifocality and advanced stage were independent risk factors of LNM. These demonstrate that PTC with LNM is more aggressive. Even in unifocal PTMC without ECS, there were still four advanced stage patients. Therefore, LNM assessment is still necessary for them. Ultrasound-guided percutaneous laser or radiofrequency ablation has come up to the clinical arena in low risk PTC treatment [41, 42]. LNM prediction guarantees the indication of such minimally invasive strategy. According to our finding, we propose that PTC patients with larger multifocal nodules or US-LNM should consider surgery. Unifocal intrathyroidal PTMC should test miR-324–5p expression in FNA washout for LNM assessment. If the expression level is higher than 2.01, surgery is recommended. Otherwise, percutaneous laser or radiofrequency ablation or following up could be considered (Figure 5).

There are some limitations to this study. This study was based on a retrospective design, and selection bias may have existed, although the US images and samples were recorded and collected prospectively. Also, the central compartment was routinely dissected, but the lateral compartment was dissected only when a LN suspicious for metastasis was detected on preoperative US. LNs that were not dissected and did not show suspicious features on US were assumed to be nonmetastatic. Moreover, this study did not include long-term follow-up. A large scale study with a prospective design and long-term follow-up is needed. The mechanism of how miR-324-5p regulates growth and metastasis in PTC also needs to be elucidated

To sum up, miR-324-5p, which is associated with LNM in PTC, promotes proliferation, migration and invasion of PTC cell. The sensitivity is highly increased when miR-324-5p is complemented with US in LNM prediction of unifocal PTMC without ECS. FNA-miR-324-5p could serve as a valuable auxiliary predictor in LNM, especially central LNM assessment of unifocal intrathyroidal PTMC.

## MATERIALS AND METHODS

### Patient selection and study design

This study was approved by the Medical Ethics Committee of Rui Jin Hospital, Shanghai, China, and all the work was conducted in accordance with the Declaration of Helsinki. All subjects provided written informed consent before surgery or FNA.

Adult patients undergoing thyroidectomy and node dissection and proven to be PTC pathologically were included in the study. Exclusion criteria were: 1. failing in miR-324-5p detection; 2. history of other malignancy; 3. history of head or neck radiation exposure in childhood; 4. history of thyroidectomy or I^131^ treatment; 5. pregnancy; 6. incomplete clinical-pathological and sonographic data.

A tentative study of 41 PTC tissues was conducted to explore the expression level of miR-324-5p between LNM (-) and LNM (+) group. Intraoperative PTC tissues were collected from inpatients at the Department of Surgery from July 2015 to July 2016. After that, miR-324-5p was further detected in 143 FNA washout specimens to assess its preoperative clinical utility. FNA washout specimens were collected from outpatients undergoing ultrasound-guided FNA (US-FNA) at the Department of Ultrasound from December 2016 to January 2017.

Pathological diagnosis was adopted as the reference standard for the verification of PTC and LNM. Disease staging was performed according to the seventh edition of American Joint Committee on Cancer (AJCC) TNM system [12]. ECS was defined as capsular invasion or extrathyroidal extension. Multifocality was defined as pathological finding of two or more PTC nodules.

### Total RNA extraction and RT-qPCR

Total RNA was extracted using TRIZOL Reagent (#15596-026, Invitrogen) according to the manufacture's protocol. For the detection of mature miRNA, miRNA was tailed and reverse transcribed using miRcute miRNA First-Strand cDNA Synthesis Kit (# KR201, TIANGEN), followed by qRT-PCR with SYBR Green chemistry using miRcute miRNA qPCR detection kit (#FP401, TIANGEN). Real-time PCR was performed in triplicate in a 384-well optical plate on the ABI ViiA™7 Sequence Detection System. The reactions were incubated at 94°C for 2 min, followed by 40 cycles of 94°C for 20 sec and 60°C for 34 sec. Analysis of relative miRNA expression data was performed using the 2^-ΔΔCt^ method [43] with the U6 small nuclear RNA as an endogenous control. Results were expressed as the amount of miR-324-5p normalized to U6 and relative to a calibrator (one LNM (-) PTC sample). The universal reversal primer was contained in the qPCR detection kit (#FP401, TIANGEN). The specific forward primers of miR-324-5p and U6 were purchased from Guangzhou RiboBio Co., Ltd. (Table 7).

**Table 7:** The sequences of miR-324-5p and U6 primer, and miR-324-5p mimics or inhibitors and their control oligonucleotides

	Sequence
hsa-miR-324-5p (forward)	5’-CGCATCCCCTAGGGCATTG-3’
U6 (forward)	5’-GCGCGTCGTGAAGCGTTC-3’
hsa-miR-324-5p mimic	Sense: 5’-CGCAUCCCCUAGGGCAUUGGUGU-3’
	Antisense: 5’-ACCAAUGCCCUAGGGGAUGCGUU-3’
hsa-miR-324-5p mimic NC	Sense: 5’-UUCUCCGAACGUGUCACGUTT-3’
	Antisense: 5’-ACGUGACACGUUCGGAGAATT-3’
hsa-miR-324-5p inhibitor	5’-ACACCAAUGCCCUAGGGGAUGCG-3’
hsa-miR-324-5p inhibitor NC	5’-CAGUACUUUUGUGUAGUACAA-3’

### Ultrasound examination and US-FNA

**Figure 6:**
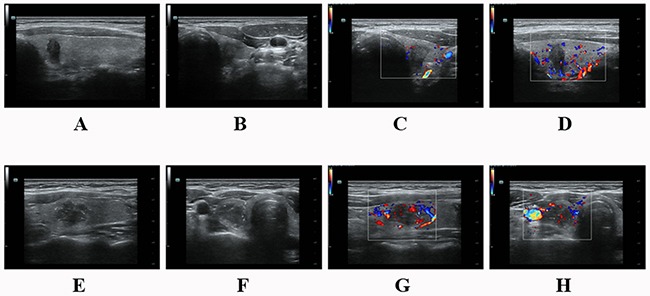
**(A-D)** A 37 year-old male PTC patient without LNM. There was a hypoechoic solid nodule on the right lobe with an irregular margin and taller than wide shape. The size was 6.9mm×10.0mm×5.8mm. No calcification was detected in the nodule. Color-Doppler showed low blood flow. **(E-H)** A 34 year-old female PTC patient with LNM. There was a hypoechoic solid nodule on the left lobe with an irregular margin and microcalcification. It located throughout the internal and external of the thyroid lobe with A/L<1 and A/T<1. The size was 14.1mm×13.2mm×18.4mm. Color-Doppler showed low blood flow. **(A&E)** Nodule throughout the anterior and posterior of thyroid lobe in longitudinal section. **(F)** Nodule throughout the internal and external of thyroid lobe in transverse section.

All grayscale and Doppler sonographic examinations were performed with 5–13MHz linear probes (ESAOTE Mylab90 and Mindray Resona 7) by experienced radiologists. US scanning involved the evaluation of the neck anatomy, including the thyroid gland, surrounding vasculature, and cervical lymph nodes. Images of each suspicious nodule or lymph node were obtained in both transverse and longitudinal orientations. All images were recorded and uploaded to a picture archiving and communication system for later retrospective analysis. The sonographic parameters of the target nodule included echogenicity, internal architecture, location (upper-, mid-, low-third, anterior, middle, posterior and throughout the anterior and posterior of thyroid lobe in longitudinal section; internal, middle, external and throughout the internal and external of thyroid lobe in transverse section), size (anteroposterior diameter (A), longitudinal diameter (L), transverse diameter (T) and the greatest dimension), shape (A/T≥1 and A/L≥1), margin (regular and irregular, the latter was specifically defined as infiltrative, microlobulated or spiculated), presence of microcalcification or not and color Doppler signal (none/low and medium/high) (Figure 6). PTMC was defined as a tumor that is 10 mm or less along its greatest dimension. Features suggesting abnormal metastatic lymph nodes included enlargement in short axis, loss of the fatty hilum, a rounded rather than oval shape, hyperechogenicity, cystic change, calcification, and peripheral vascularity [12]. For patients with multiple nodules, only the most suspicious nodule corresponding to the pathological outcome was included. The time interval between US and operation for PTC tissue was within one month with the median time of 8 days. All the sonographic features were assessed by one researcher on the premise of not knowing the pathological results.

US-FNA was performed by interventional radiologists, using US-guided technology with 25G or 22G needle and 5ml syringe. All interventional radiologists had undergone standard training. Patients were placed in a supine position with the neck hyperextended. Each nodule was punctured at least twice and aspired for five to eight times for each smear.

### Cell culture and transfectoin

The human KTC1 cell line was purchased from Chinese Academy of Science. KTC1 cells were cultured in RPMI 1640, MEM NEAA, GlutaMAX™ and Sodium Pyruvate 100 mM Solution (Gibco, 87:1:1:1) medium supplemented with 10% of FBS (Gibco), 100 U/mL penicillin, and 100 μg/mL streptomycin. Cells were incubated at 37°C in a humidified atmosphere of 5% CO2.

MiR-324-5p mimics or miR-324-5p inhibitors and their control oligonucleotides were synthesized by Shanghai Genepharma Co., Ltd. The sequences are listed in Table 7. A day before transfection, cells were plated at a density of 1.5×10^5^ cells/well into 6-well plates. After culturing for 24h when the cell confluence was about 70%, transfection was conducted with Lipofectamine 2000 (Invitrogen, USA) following the manufacturer's protocol. Transfection effect of miR-324-5p mimic and inhibitor in KTC1 cell line was illuminated in Supplementary Figure 1. Functional assays were conducted 24h-96h after transfection. Transfection efficiencies were evaluated by qRT-PCR.

### Cell proliferation assay

Cells were seeded in 96-well plates at approximately 2000 cells/well and cultured in the appropriate medium. Numbers of viable cells were quantified using cell counting kit 8 (Dojindo Molecular Technologies, Japan) at 24 h, 48 h, 72 h, 96 h by measuring OD450 with TECAN infinite M200 plate reader.

### Colony formation assay

Cells were seeded into six-well plates with 1000 cells/well in 2ml culture medium and incubated at 37°C in a humidified atmosphere of 5% CO2. The cultured medium was replaced every the other day. After 10 days in culture, the medium was removed and the cells were washed twice with PBS. Finally, the cells were stained with crystal violet for 30 minutes at room temperature, washed again and photographed. Three independent experiments were performed.

### Flow cytometry

The apoptosis assay was performed with an Alexa Fluor® 488 Annexin V/Dead Cell Apoptosis Kit (Life Technologies, USA). The cells were suspended in 100 μL binding buffer with 5 μL Annexin V and 1 μL propidium iodide and were incubated for 15 minutes in the dark. Then, binding buffer was added to 400 μL and the cells were resuspended. For the cell cycle analysis, the cells were fixed in 75% ethanol overnight at 4°C. They were then stained using a Cell Cycle and Apoptosis Analysis Kit (Beyotime Biotechnology, China) according to the manufacturer's instructions. Cell apoptosis and cell cycle were tested and analyzed by a Gallios Flow Cytometer (Beckman Coulter, USA). All experiments were performed independently three times.

### Transwell assay

Transwell chambers (Millipore, USA) were used in the migration and invasion assays. Matrigel (BD Biosciences, USA) was used to coat the top side of the membrane used in the invasion assay. Then, 100 μL serum-free medium and 1×10^5^ cells were added to the upper chamber, while 500 μL medium with 5% FBS was added to the lower chamber. The chambers were maintained at 37°C and 5% CO2 for 24 h, followed by removal of cells inside the upper chamber with cotton swabs. Migrated or invaded cells on the membrane bottom-surface were fixed in methanal for 15 minutes and stained with crystal violet for 30 minutes. Cells adhering to the under surface were calculated and photographed using an inverted phase contrast microscope. All experiments were performed in triplicate.

### Scratch assay

Cells were seeded on six well plates with RPMI 1640 containing 10% FBS and grown to monolayer confluency. Each monolayer was scratched with a sterile pipette tip. The wound healing procedure was observed for 24 h, and images were photographed at 0 h and 24 h.

### Statistical analysis

The statistical analysis was performed with IBM SPSS Statistics (version 23.0). Descriptive statistics were presented as mean ± SD for normal distribution data or quartile for non-normal distribution data; categorical variables were presented as proportions and frequencies. K-S test was used to determine the normality of variable. Chi-square test or fisher's exact test was used to analyze the categorical data. Two-tailed Student's t test or Mann Whitney U test was performed to compare the descriptive statistics as appropriate. ROC analysis was performed for the capacity of different variables to discriminate LNM and AUC value was calculated. Data yielding the maximal sum of sensitivity and specificity was set as the optimal cut-off value from ROC. Multivariate logistic regression analyses were performed to determine the independent factors of LNM (selectin method was Forward LR). Odds ratios (ORs) with 95% confidence intervals (CIs) were calculated. P<0.05 was considered statistically different in all tests.

## SUPPLEMENTARY MATERIALS FIGURE


