# Supplementary material for: MiR-324-5p assists ultrasonography in predicting lymph node metastasis of unifocal papillary thyroid microcarcinoma without extracapsular spread
Source: Oncotarget. 2017 Jul 31;8(48):83802–16. doi: 10.18632/oncotarget.19717 (PMC5663556; doi:10.18632/oncotarget.19717)
Supplement: Supplementary file 1 [file oncotarget-08-83802-s001.pdf]

# MiR-324-5p assists ultrasonography in predicting lymph node metastasis of unifocal papillary thyroid microcarcinoma without extracapsular spread

## SUPPLEMENTARY MATERIALS

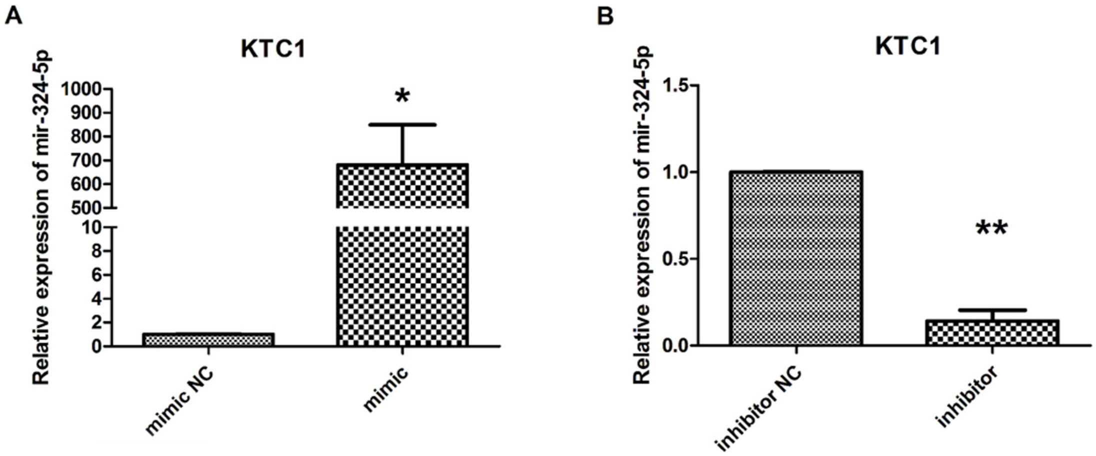


**Supplementary Figure 1:** Transfection effect of miR-324-5p mimic **(A)** and inhibitor **(B)** in KTC1 cell line. Data are expressed as means ± SD of three independent experiments. *P<0.05, **P<0.01.
